# Quantitative approach for the risk assessment of African swine fever and Classical swine fever introduction into the United States through legal imports of pigs and swine products

**DOI:** 10.1371/journal.pone.0182850

**Published:** 2017-08-10

**Authors:** Diana María Herrera-Ibatá, Beatriz Martínez-López, Darla Quijada, Kenneth Burton, Lina Mur

**Affiliations:** 1 Department of Diagnostic Medicine/Pathobiology, College of Veterinary Medicine, Kansas State University, Manhattan, KS, United States of America; 2 Center for Animal Disease Modelling and Surveillance (CADMS), University of California Davis, Davis, CA, United States of America; 3 National Agricultural Biosecurity Center, Kansas State University, Manhattan, KS, United States of America; Nanjing Agricultural University, CHINA

## Abstract

The US livestock safety strongly depends on its capacity to prevent the introduction of Transboundary Animal Diseases (TADs). Therefore, accurate and updated information on the location and origin of those potential TADs risks is essential, so preventive measures as market restrictions can be put on place. The objective of the present study was to evaluate the current risk of African swine fever (ASF) and Classical swine fever (CSF) introduction into the US through the legal importations of live pigs and swine products using a quantitative approach that could be later applied to other risks. Four quantitative stochastic risk assessment models were developed to estimate the monthly probabilities of ASF and CSF release into the US, and the exposure of susceptible populations (domestic and feral swine) to these introductions at state level. The results suggest a low annual probability of either ASF or CSF introduction into the US, by any of the analyzed pathways (5.5*10^−3^). Being the probability of introduction through legal imports of live pigs (1.8*10^−3^ for ASF, and 2.5*10^−3^ for CSF) higher than the risk of legally imported swine products (8.90*10^−4^ for ASF, and 1.56*10^−3^ for CSF). This could be caused due to the low probability of exposure associated with this type of commodity (products). The risk of feral pigs accessing to swine products discarded in landfills was slightly higher than the potential exposure of domestic pigs through swill feeding. The identification of the months at highest risk, the origin of the higher risk imports, and the location of the US states most vulnerable to those introductions (Iowa, Minnesota and Wisconsin for live swine and California, Florida and Texas for swine products), is valuable information that would help to design prevention, risk-mitigation and early-detection strategies that would help to minimize the catastrophic consequences of potential ASF/CSF introductions into the US.

## Introduction

African swine fever (ASF) and Classical Swine Fever (CSF) are animal diseases notifiable to the World Organisation for Animal Health (OIE) [1] that have devastating impact in the affected countries. Both diseases cause hemorrhagic fever syndromes in swine, with similar clinical presentation and high mortality rates. Nevertheless, they are caused by viruses classified in different families: ASF virus (ASFV) is a complex DNA virus unique member of the *Asfarviridae* family [2], whereas CSF is caused by a RNA virus of the genus *Pestivirus*, family *Flaviviridae* [3]. ASF and CSF exclusively affect swine, domestic and wild, and can be transmitted by either direct contact between pigs or indirect transmission, primarily through the ingestion of infected products and contact with other contaminated fomites [2, 3]. ASFV is also transmitted by soft ticks (*Ornithodoros spp*.) [4], being *O*. *moubata* the main vector in Africa and *O*. *erraticus* in the Mediterranean [5].

ASF was first described in Africa in 1921, which remains endemic in most of Sub-Saharan countries [6]. However, the disease has not been uniquely restricted to the African continent. Between the 1960s and the 1990s ASF was present in the Iberian Peninsula causing sporadic outbreaks in other countries of Europe, the Caribbean and South America (Brazil). In 1995, the disease was successfully eradicated from all those territories except from the Italian island of Sardinia where is still endemic. The global epidemiological situation of ASF drastically changed after the introduction of ASFV for the first time in Georgia in 2007 [7]. From there, ASFV efficiently spread to extensive areas of the Caucasus region, affecting southern and western Russia [8], Belarus and Ukraine, until it finally reached the European Union (EU) in 2014. By 2017, ASF is known to be present in the Russia, Belarus, Ukraine, Baltic countries (Estonia, Latvia and Lithuania), Poland and Moldova [9]. The continuous spread of the disease towards western regions with continuous cases of ASF in wild boar and domestic pigs, reflect the lack of success of control programs in the area and the potential threat for the worldwide swine industry due to the absence of vaccine [10].

While ASF had been traditionally associated with the African continent (and Sardinia) until its re-introduction in Europe, CSF has been much wider distributed causing important problems around the world for decades [11]. Notable are the losses originated in many European countries during the 1990s after the vaccination banning policy [12] and the widespread of genotypes 2 and 3 in China [13]. Despite the availability of vaccines, by 2017 CSF is still endemic in many parts of Asia, South America, and some Caribbean islands close to the United States (US) such as Cuba, Haiti and Dominican Republic [14].

The United States of America (US) is the world's third-largest producer and consumer of pork, and the world’s largest pork exporter, with an average of 20% of pork annually produced in the US being exported [15]. Therefore, it is essential to develop prevention strategies and early-warning systems based on risk analysis to reduce the likelihood of introduction of Transboundary Animal Diseases (TADs). ASF has never occurred in the US and no antiviral treatment nor vaccine is available, nor is expected to be available in the short term, so disease prevention is essential as control measures are exclusively based on stamping out policies. In contrast, for CSF virus (CSFV) there are several effective vaccines available in the market [16], even in oral bait format, which have been successfully used in wildlife through mass vaccination campaigns [17]. However, as the US already experienced in the past, the control and eradication of CSF poses important control challenges and devastating economic impacts for the swine industry (i.e. the approximate cost of controlling CSF in the US in 1978 was $140 million).

ASF and CSF represent a risk for the global swine industry, and important economic consequences are expected if they were introduced in swine production countries as the US [18, 19]. Therefore, the objective of the present study was to evaluate the risk of both, ASF and CSF introduction into the US at state level by legal imports of live pigs and swine products in a monthly basis. The results of these assessments will help to identify the potential routes, locations and times when the country livestock population is at higher risks and when preventive measures should be implemented to avoid the introduction of ASF and CSF into the US. The results obtained will be incorporated in an online platform (Disease BioPortal™ accessible at http://bioportal.ucdavis.edu/) to facilitate their visualization and analysis, and the quantitative approach developed here will serve to develop a template for the assessment of the potential introduction of other TADs.

## Materials and methods

Four quantitative stochastic risk assessment models were developed to estimate the monthly probabilities of ASF and CSF introduction into the US by legal imports of live pigs and products during the high risk period (i.e., period of time from the infection in the country of origin to the detection and notification). Specifically, the four models addressed the following event pathways: i) risk of ASF being introduced by legal imports of live pigs, ii) risk of ASF introduction by legal imports of swine products, iii) risk of CSF introduction by legal imports of live pigs and, iv) risk of CSF being introduced by legal imports of swine products.

Finally, those four models were combined to estimate i) the probabilities of ASF introduction and ii) the probability of CSF introduction by any of the analyzed pathways. These two probabilities were finally combined to estimate the probability of either, ASF or CSF, introduction into the US by legal importations of pigs or products. All models were developed in @RISK 7.5 (Palisade Corporation, Newfield, NY, USA) on Microsoft Excel 2007^®^ and run 1,000 iterations using the Monte-Carlo sampling method.

*Note: In order to simplify the text and avoid redundancies, the sentences, parameters and probabilities that apply for both diseases’ models (the model developed for ASF and the model for CSF), were referred as “ASF/CSF”. For example “the probability of ASF/ CSF introduction into the US by the legal imports of live pigs” is read as “the probability of ASF introduction into the US and the probability of CSF introduction into the US”. It is important to differentiate this notation with the probability of both events (ASF and CSF) occurring at the same time (P_ASF_∩P_CSF_).

### Models specifications

**Fig 1:**
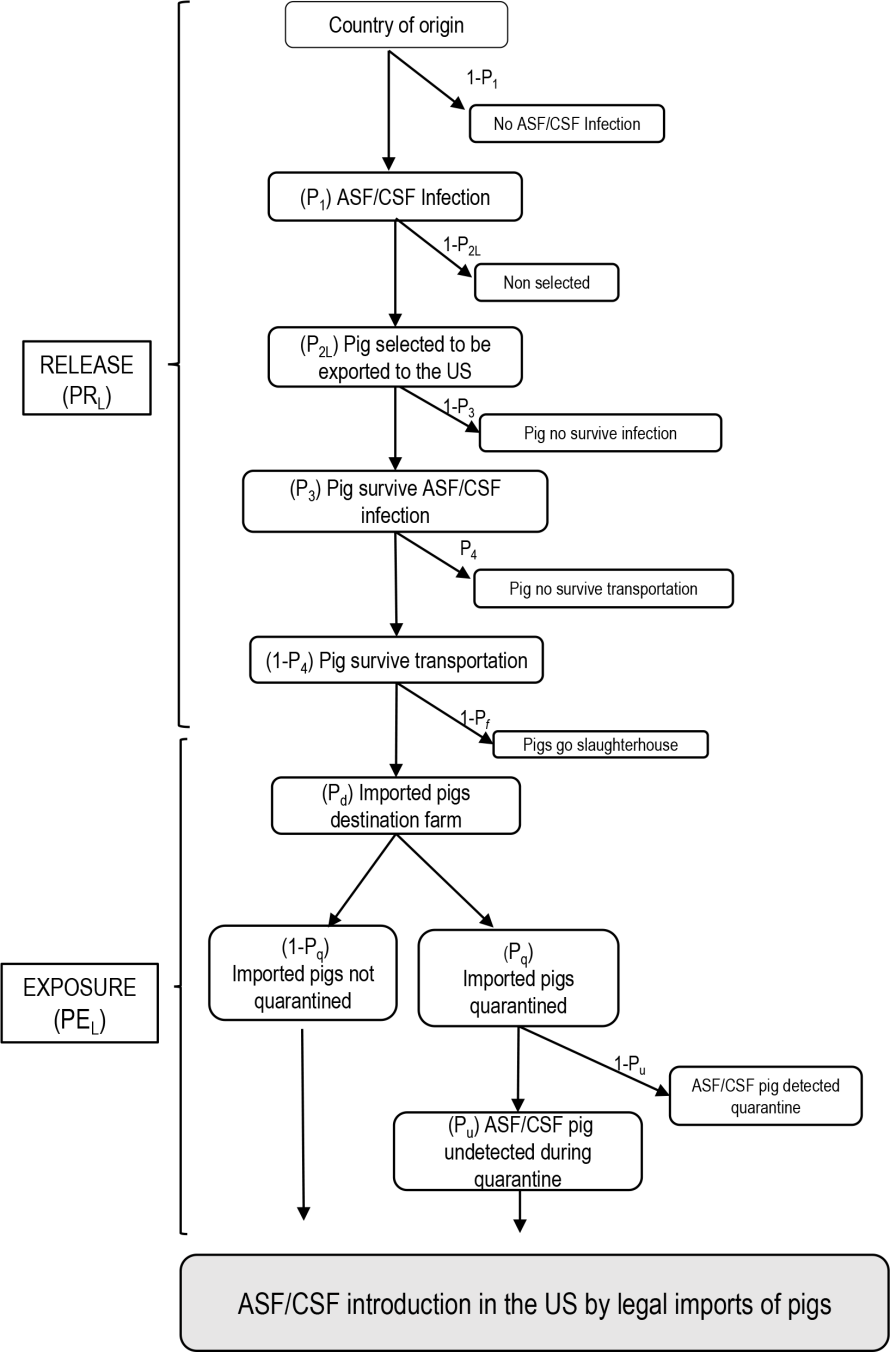
Event tree of ASF /CSF introduction into the US by the legal imports of live pigs.

**Fig 2:**
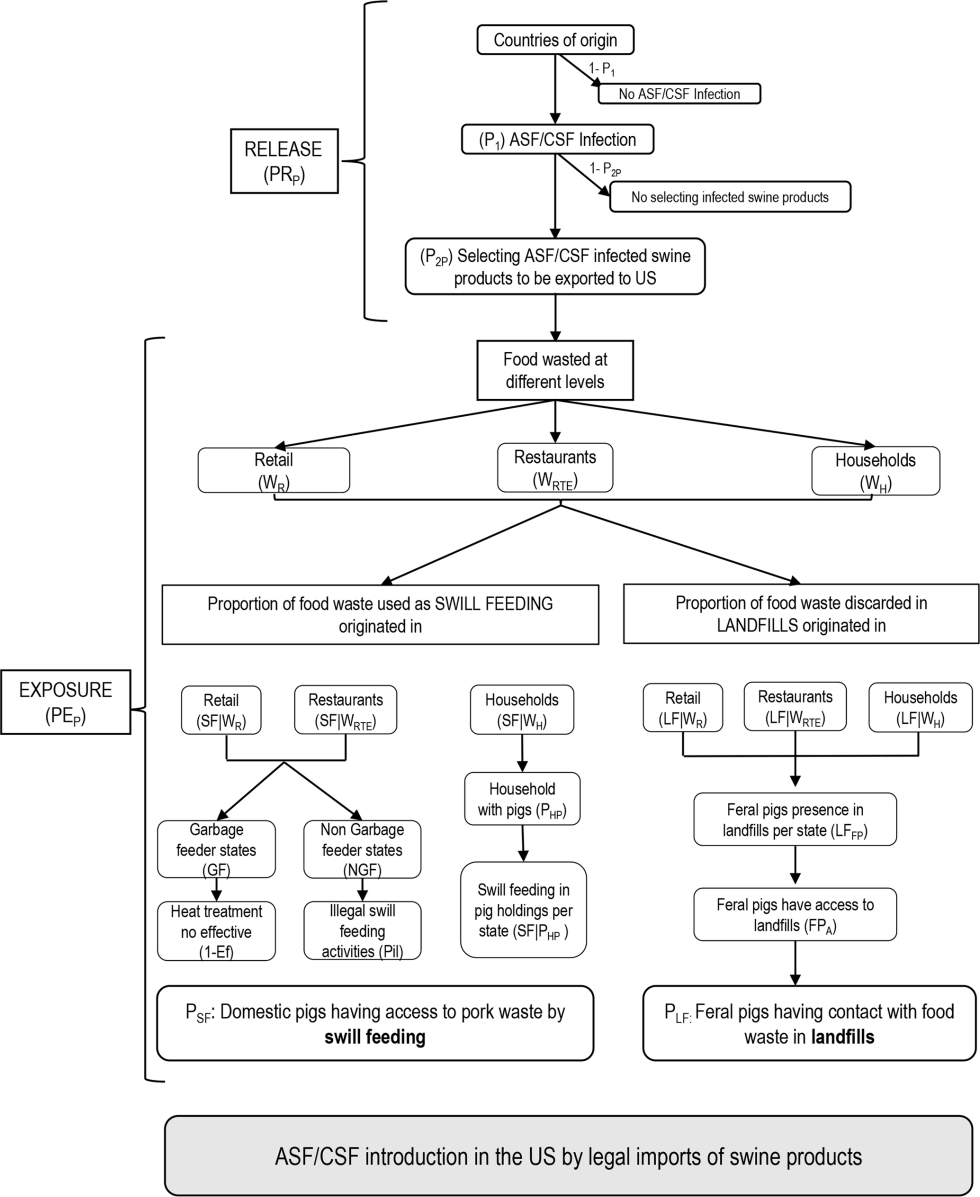
Event tree of ASF/ CSF introduction into the US by the legal imports of swine products.

Based on OIE guidelines [20], the risk analysis for the introduction of pathogens through imports is divided in three steps: entry (formerly and in the present study called release [21]), exposure and consequence assessments. The risk assessment models developed in this study assessed the probability of ASFV and the probability of CSFV being released into the US and the subsequent exposure of the US susceptible populations (domestic pigs and feral pigs). The final probability for each pathway was calculated as follows:

$$P_{F}=P_{R}*P_{E}$$

where P_F_ is the final probability, P_R_ is the probability of ASF/CSF being released into the US, and P_E_ is the probability of exposure. The event trees depicted in Figs 1 and 2 summarize the structure and chain of events of the risk pathways of the legal imports of live pigs and swine products, respectively. Table 1 includes detail information of the countries of origin and the imports to the US, while Tables 2, 3 and 4 include detail information about the parameters used to feed the four models. Specifically, Table 2 includes the parameters used in the release and exposure assessment of the risk associated with live pigs imports, while the information for the risk of swine products imports was split into release (Table 3) and exposure (Table 4).

**Table 1:** Pig and swine products imports to the US and disease status of the countries of origin.

	Pigs		Swine products		Disease Presence	
	Heads	%	Kg	%	ASF	CSF
**Austria**	34				N	N
**Australia**			2.9E+04		N	N
**Brazil**			2.4E+05		N	N (09)
**Canada**	4.9E+07	99.9	2.7E+09	81.7	N	N
**Cayman Islands**			2.1E+04		N	N
**Chile**			1.4E+07	0.4	N	N
**China**			5.2E+04		N	Y
**Colombia**			2.5E+04		N	Y
**Croatia**			5.0E+05		N	N (08)
**Denmark**			2.9E+08	8.5	N	N
**Ecuador**			2.3E+03		N	Y
**Finland**			6.9E+06	0.2	N	N
**France**			1.1E+05		N	N
**Germany**			2.4E+06		N	N
**Hungary**			1.8E+06		N	N (09)
**Ireland**	1273		2.9E+07	0.9	N	N (09)
**Italy**	21		4.3E+07	1.3	R	N
**Mexico**			5.3E+07	1.6	N	N
**Netherlands**	18		2.9E+07	0.9	N	N
**New Zealand**			4.1E+04		N	N
**Philippines**			8.0E+01		N	N
**Poland**			1.1E+08	3.4	Y	N
**San Marino**			2.0E+03		N	N
**Spain**	180		1.3E+07	0.4	N	N
**Sweden**			9.6E+05		N	N
**United Kingdom**	15		2.3E+07	0.7	N	N

Total volume of pigs (heads) and swine products (kg) imported to the US from 2008 to 2015 and the percentages by country of origin. The status of ASF and CSF of these countries is noted as follows (N: No disease present from 2015, Y: Disease present, and R: restricted to certain territories). When outbreaks have occurred from 2005 but the disease is not present at the moment, the date of the last occurrence was included in parenthesis.

**Table 2:** Description of input parameters and probabilities used in the quantitative models for the release and exposure assessment of the risk of ASF / CSF introduction into the US through legal imports of live pigs.

Notation	Definition	Parametrization	Source		Values
P_1_	Probability of ASF^a^/CSF^b^ infection in the country of origin	Free countries: Pert (min, most likely, max)	Free- countries: SI model results		
P_2L_	Probability of selecting an ASF^a^/CSF^b^ infected pig from country *c* before the detection of the infection	Beta (α_1_, α_2_) • α_1L_ = NI + 1 • α_2L_ = N_o_-(NI + 1)	• NI = Ou*To*Hp • No = pig population in c		
O_u_	Number of ASF^a^/CSF^b^ undetected outbreaks before official notification	Pert (min, most likely, max)	^a.^ Outbreaks in Europe (2007–2016) [14] ^b.^ World outbreaks (2006–2016) [14]		^a.^ Pert (1, 1.28, 6) ^b.^ Pert (1, 2, 3)
T_o_	Average herd size in country *c*	Normal = N_o_/S_o_	[24]		
N_o_	Pig population in country *c*	Normal (0078, σ)	[14] [24]		
S_o_	Number of pigs establishments in country *c*	Normal (μ, σ)	[14]		
H_p_	Intra-herd prevalence ^a^ASF/^b^CSF	Pert (min, most likely, max)	^a.^ Data from outbreaks in Europe (cases/susceptible) [14] ^b.^ [30]		^a.^ Pert (0.05, 0.15, 0.32) ^b.^ Pert (0.05, 0.4, 1)
P_3_	Probability of pigs surviving to ^a^ASF/^b^CSF infections	Pert (min, most likely, max)	^a^[31];^b^[32]		^a.^ Pert (0.05, 0.2, 0.8) ^b.^ Pert (0, 0.2, 0.4)
P_4_	Probability of pigs not surviving transportation	Pert (min, most likely, max)	[33]		Pert (0.0005,0.0027,0.092)
PR_L_	Probability of release of at least one ASF/CSF infected domestic pig by legal imports from the country of origin (*c*) into a state of the US (*s*)	$PR_{L}=1-{\left(1-p_{csL}\right)}^{n_{csL}}$			
p_csL_	Probability that an ASF^a^/CSF^b^ infected pig from country *c* arrives to the US state *s* during month *m*	Binomial (n, p)	n = n_csL_; p = P_1_*P_2L_*P_3_*P_4_		
n_csL_	Imports of live pigs from country *c* to the US by state *s* (2008–2015)	Normal (μ, σ)	[22][23]		
PE_L_	Probability of a domestic pig in the US getting an effective contact with a legally imported infected pig	*PE*_*L*_ = *P*_*d*_ * [(1−*P*_*q*_) + (*P*_*q*_ * *P*_*u*_)]			
P_d_	Probability of imported pigs have a farm destination	Normal (μ, σ)		USDA APHIS dataset from [32]	Normal (0.45, 0.04)
P_q_	Probability of imported pigs going through quarantine	Beta (α_1_,α_2_)		Assumption based on [34]	Beta (68.7, 4.6)
P_u_	Probability that an ASF^a^/CSF^b^ infected pig is undetected during quarantine	Beta (α_1_, α_2_)		Expert Opinion (Luis Romero) [35]	Beta (1.3, 34.2)

**Table 3:** Description of input parameters and probabilities used in the quantitative models for the assessment of the risk of ASFV/ CSFV release into the US through legal imports of swine products. Note: the information about the inputs marked with* is listed in Table 2.

Notation	Definition	Parametrization	Source	Values
P_1_	Probability of ASF^a^/CSF^b^ infection in the countries of origin	1. Infected countries: • P_1_ = 1- Exp (-t*λ) • t: time interval (one month) • λ = a/b where a: number of outbreaks in domestic swine, and b: the total number of months of the study period 2. Free countries: Pert (min, most likely, max)	1. [14] 2. Free-countries: SI model	*Examples*: 1.^a^ Poland (0.47) 1.^b^ China (0.61) 2.^a^Canada (0, 0.001, 0.002) 2.^b^Canada (0, 0, 0.005)
P_2P_	Probability selecting ASF^a^/CSF^b^ infected meat	Beta (α_1p_, α_2p_) • α_1p_ = Q_IM_+1 • α_2p_ = N_m_-(Q_IM_ + 1)	• Q_IM_ = NI*P_m_*M_*p*_ • NI = Ou*To*Hp*	*Examples*: ^a.^ Canada 9.53*10–9 ^b.^ Canada 5.19*10–8
P_m_	Probability ASF^a^/CSF^b^ infected pig being transformed into meat		P_m_ = P_3_*·(1-P_4_)*·P_m_*·*P_us_	^a.^ ASF 9.74*10–4 ^b.^ CSF 2.78*10–3
P_sm_	Probability of a pig going to slaughterhouse during a specific month	Normal (μ, σ)	[36]	Normal (0.18, 0.02)
P_us_	Probability of an infected pig being undetected in slaughterhouse	Beta (α_1_, α_2_)	Expert Opinion (Luis Romero)[35]	Beta (1.34, 34.17)
M_p_	Average kg meat obtained from pig	Normal (μ, σ)	[24]	Normal (60.9, 2.3)
N_m_	Total meat production country *c*	Normal (μ, σ)	[24]	Normal (1941995, 34933.4)
PR_P_	Probability of release of at least kg of swine products infected with ASFV/CSFV by legal imports from the country of origin (*c*) into a state of the US (*s*)		$PR_{P}=1-{\left(1-p_{csP}\right)}^{n_{csP}}$	
p_csP_	Probability that an ASF^a^/CSF^b^ infected product from country *c* arrives to the US state *s* during month m	Binomial (n,p)	n = n_csP_; p = P_1_*P_2P_	Canada to Alabama, January: ^a^1.78*10^−8; b^6.37*10^−8^
n_csP_	Imports of swine products (kg) from country *c* to the US by state *s* (2008–2015)	Normal (μ, σ)	[22] [23]	Example: Imports from Canada to Alabama during January: Normal (6529.21, 1072.88)

**Table 4:** Input parameters and probabilities used in the quantitative models for the assessment of the exposure of US swine populations to imported swine products (PE_P_).

Notation	Definition	Parametrization	Source	Values
W_R_	Proportion food waste in the retail sector	Normal (μ, σ)	[51] [52]	Normal (0.09, 0.02)
W_C_	Proportion of food waste at consumer level (restaurants and households)	Normal (μ, σ)	[53][54]	Normal (0.23, 0.06)
C_RTE_	Proportion of pork consumed in restaurants vs households	Normal (μ, σ)	[55][56]	Normal (0.20, 0.03)
SF_R &_ SF_RTE_	Probability of domestic pigs having access to pork waste originated in retail (R) or restaurants (RTE) by swill feeding		• SF_R_ = W_R*_ SF|W_R_*(GF*((1- Ef) + P_il_)) • SF_RTE_ = W_RTE*_ SF|W_RTE_*(GF*((1- Ef) + P_il_))	
SF|W_R_	Proportion of food waste from retail used in swill feeding	Pert (min, most likely, max)	[41] [45]	Pert (0.05, 0.11, 0.15)
SF|W_RTE_	Proportion of food waste from restaurants used in swill feeding	Normal (μ, σ)	[41][45]	Normal (0.02, 0.002)
GF	States of the US where garbage feeding is allowed	Boolean variable	[43]	(0, 1)
Ef	Efficacy of heat treatment of food waste used to feed animals	Beta (α_1_, α_2_)	[57]	Beta (99.7, 6.2)
P_il_	Probability of illegal swill feeding activities in states where garbage feeder is not allowed	Beta (α_1_, α_2_)	Assumption based on [57]	Beta (99.7, 6.2)
SF_H_	Probability of domestic pigs having access to pork waste originated in households (H) by swill feeding		SF_H_ = W_C_*(1-C_RTE_)* SF|WH* P_HP_*(SF|P_HP_).	
SF|W_H_	Proportion of food waste from households used in swill feeding	Normal (μ, σ)	[42]	Normal (0.048, 0.005)
P_HP_	Probability of a household in the US having pigs presence	Pert (min, most likely, max)	[47][58]	Pert (0.004, 0.011, 0.013)
SF|P_HP_	Probability of pigs holdings using swill feeding per state (based on size farms)	Normal (μ, σ)	[48]	Example: California (0.23, 0.02)
P_LF_	Probability of feral pigs having contact with food waste in landfills	PLF = (W_R_*LF_R_+ R_RTE_*LF_RTE_ + W_H_*LF_H_)* FP_A*_ LF_FP_		
LF|W_R_	Proportion of food waste from retail sector discarded in landfills	Normal (μ, σ)	[41][45]	Normal (0.57, 0.1)
LF|W_RTE_	Proportion of food waste from restaurants discarded in landfills	Normal (μ, σ)	[45]	Normal (0.84, 0.1)
LF|W_H_	Proportion of food waste from households disposed in landfills	Normal (μ, σ)	[45]	Normal (0.95, 0.1)
LF_FP_	Probability of feral pigs presence in landfills per state	Pert (min, most likely, max)	Spatial analysis (Section 2.1.2)	*Example* Alabama Pert (0.0005, 0.84, 0.87)
FP_A_	Probability that feral pigs have access to landfills	Pert (min, most likely, max)	Assumption	Pert (0.05,0.1,0.2)

### Release assessments

The probability of release (P_R_) of at least one ASF /CSF infected domestic pig or kilogram (kg) of swine product by legal imports from the country of origin (*c*) into a state of the US (*s*), was modeled following a binomial process of the form:

$$P_{R}=1-{\left(1-p_{cs}\right)}^{n_{cs}}$$

Where *n*_*cs*_ refers to the number of pigs or kilograms (kg) of swine products (depending on the pathway assessed) legally imported from the origin country *c* to the destination states *s*; and *p*_*cs*_ is the probability that an ASF/CSF infected animals or products (including chilled, frozen and smoked meat, fats and offal from swine) arrive from origin country *c* to destination states *s*.

The information on imported live pigs and swine products (*n*_*cs*_*)* was obtained from the USDA Global Agricultural Trade System (GATS) [22] and US Census Bureau USA trade [23] from 2008 to 2015. GATS database contained the quantities and value ($) of imported live animals (number of pigs) and products (kg) by month and country of origin, but aggregated for the whole US. On the other hand, the US Census Bureau database included the value ($) of imports per month, country of origin and US states of destination. Both databases were combined to estimate the quantity (number of pigs and kg of products) imported per month from each country of origin to each state of the US. More detailed information on the import data and the countries of origin can be found in Table 1.

The probability *p*_*cs*_ was calculated as the product of different conditional probabilities as follow:

$$p_{cs}=\prod_{i=1}^{x}P_{i}$$

Specifically, the probability of release for the legal importations of live pigs (p_csL_) was calculated as the product of four conditional probabilities (Fig 1). The probability of ASF / CSF infection in the origin country (P_1_); the probability of selecting a pig infected with ASFV / CSFV in country c (P_2L_); the survival probability of ASF/CSF infection (P_3_); and the probability that infected pig survives transportation from country c to destination s (1-P_4_). Due to the lack of information related with carrier animals (their prevalence, transmission capacity, etc.), only apparent clinical stages of the disease were considered in the models.

The probability of release for the legal importation of products (p_csP_) (Fig 2) was estimated as the product of two conditional probabilities: P_1_ (probability of ASF /CSF infection in the origin country, following the same structure of the live pig models) and P_2P_ as the probability of selecting a kilogram of swine products infected with ASFV/CSFV in country c.

#### Probability of infection in the countries of origin (P1)

The probability of infection in the origin country *c* was parameterized differently depending on the status of the diseases in the country of interest. For those countries currently infected by ASF / CSF, an exponential function was used to estimate the probability of at least one outbreak in the considered time interval with the following form:

$$P_{1}=1\text{-}\;\mathrm{Exp}\;\left(\text{-}t*\lambda \right)$$

where *t* is the time interval (one month) and λ is the mean number of disease outbreaks per month obtained from historical outbreak information [14] estimated as λ = a/b. Being *a* the total number of outbreaks in domestic swine and *b* the total number of months of the study period.

For those countries with no records of ASF/ CSF presence for the last 10 years, the probability of infection was estimated through a compartmental Susceptible (S)-Infected (I) model that considers the pig trade network and the disease status of trading countries. Specifically, the model used data on world pig trade from the latest year available (2013) [24], and disease status of the trading countries based on OIE- WAHIS country information (presence or absence of ASF /CSF)[14]. Two models were built, one where the seed of infection was randomly allocated in the ASF positive countries, and other one where simulations start in CSF positive countries. In both cases, we created the directed adjacency matrixes following the trade directionality (from exporter to importer). A high risk period of 30 days and two levels of potential connection degrees between the trading countries were assumed for all models. Several scenarios with different transmission rates (pT) were assayed for each disease including 1%, 2.5%,5% and 10% for ASF [25]; and 0.4%, 6% and 12% for CSF [26]. The SI models were built in R software [27] using the packages *igraph* [28] and *sna* [29]. Each scenario was run with 1,000 simulations. The proportion of times each receiving country got infected during the total simulations was considered as the risk of infection in the origin country. The minimum, median and maximum values of the risk obtained in the different model scenarios were used to parameterize the Pert distributions (min, most likely, maximum) for the risk of infection in the countries of origin (see examples in Tables 2 and 3).

#### Probability of selecting an infected live pig (P_2L_) or product (P_2P_) in country c before the detection of the diseases

In the models assessing the risk associated with imports of live pigs, the probability of selecting an infected live pig in origin country *c* was modeled using a beta distribution defined by the following parameters: α_1L_ as the number of estimated infected pigs prior to ASF/CSF detection in the country *c*, and α_2L_ as the total number of pigs in country *c*. The number of infected pigs in country *c* (NI) was estimated as the product of three independent parameters: i) undetected outbreaks (Ou) during the high risk period in country *c* [14], ii) average pig herd size in origin country (To) [24], and iii) intra-herd prevalence (Hp) for ASF/ CSF [14] (Table 2). For the estimation of the disease-related parameters (Ou and Hp), in the case of ASF, only the reports from recent European outbreaks were considered, as the reports from Africa frequently lack the essential data required. For the calculations of Ou, we counted the number of outbreaks occurring in newly affected countries between the first event and the date of first report (high risk period), whereas the intra-herd prevalence was estimated per event considering the number of cases vs the number of susceptible animals present in the outbreak.

In the models developed to estimate the risk associated with imports of swine products the probability of selecting infected products from the country of origin *c* was also parameterized with a beta distribution characterized by two parameters: α_1P_ as the quantity (kg) of potentially infected swine products in the country *c*, and α_2P_ as the quantity (kg) of swine products produced (N_P_) in the country *c*. The quantity of infected products in the origin country *c* (QIM) was estimated by the product of three parameters: NI) estimated number of infected pigs in the country of origin *c*, P_m_) probability of an ASF/CSF infected pig being transformed into meat, and M_P_) the average weight of products (kg) obtained per slaughtered pig (Table 3).

The number of infected pigs (NI) was calculated identically to the live pig models, as the product of Ou* Hp *To. The probability of an ASF/CSF infected pig being transformed into meat (P_m_), was estimated as follows: P_m_ = P_3_*(1-P_4_)*P_sm_*P_us_

where P_3_ is the probability of a pig surviving ASF / CSF infection, P_4_ is the probability of a pig not surviving the transportation, P_sm_ is the probability of a pig being slaughtered during a specific month, and P_us_ as the probability ASF/ CSF infected pigs being undetected during the clinical checks performed in the slaughterhouse. The P_sm_ was estimated using historical data of pig census and the monthly proportion of pigs sent to slaughter in a variety of countries. Data from the EU countries was used for that purpose [36], assuming that all type of pig production systems are represented in them.

### Exposure assessment

The probabilities of susceptible populations (domestic and feral pigs) in the US getting in contact with the viruses strongly differ depending on the type of infected matrix (live pigs or swine products). Therefore, the exposure assessments for both pathways were analyzed differently and explained separately below.

#### Probability of exposure of US domestic swine to imported domestic pigs (PE_L_)

The probability of a domestic swine in the US getting an effective contact with a legally imported infected pig (PE_L_) was estimated as following:

$$PE_{L}=P_{d}*[(1-P_{q})+(P_{q}*P_{u})]$$

Where P_d_ is the probability of an imported pig having a farm destination, P_q_ as the probability of imported pigs being quarantined, and P_u_ as the probability of infected pigs being undetected during quarantine. P_q_ was parametrized as a Beta distribution using BetaBuster [37] considering a most likely value of 0.95 and 90% confidence of the probability being higher than 0.9. These values were assumed by the authors based on US legislation on quarantine procedures [34] and certain degree of uncertainty due to non-proper compliance of the legislation. Considering the similarities in the clinical presentation of ASF and CSF, we assumed the same probability of non-detection for both diseases, and consequently, the same probability of exposure to potentially infected imported pigs. All the information related with these probabilities is included in Table 2.

The potential contact of imported domestic pigs with feral pigs in the US was not included in the model, as considering the characteristics of swine pigs imported (99.9% are feeder pigs coming from Canada [38]), their most probable destination will be a finishing facility with high biosecurity standards and no potential contact with feral pigs.

#### Probability of exposure to imported swine products into the US (PE_P_)

The detailed information about the inputs and calculations used to estimate the probability of exposure to imported swine products into the US can be found in Table 4 and Fig 2. The swine products legally imported into the US could be discarded and become food waste either at the selling point if not sold on time (called retail herein), or at the consumer level (restaurants and house-holds). Other sources of food waste are the institutions, hospitals, colleges or prisons. However, they contribution to the total food waste generated in the US is limited (between 5.4 and 8%)[39, 40]. Therefore, considering that, and the absence of detailed information for the management of the waste generated in this sector, only three categories were included in the analysis. Specific statistics of the proportion of food and pork wasted at each level were used to estimate the probability of swine products not being consumed at the three different sectors of the consumption chain analyzed (W_R_, W_RTE_ and W_H_ for the retail, restaurants and house-holds, respectively). The probability of pork being discarded at house-hold level (WH) was estimated as the product of the proportion of food being wasted at consumer level (W_C_) by the proportion of pork consumed at houses instead of restaurants (1-C_RTE_). The opposite applies for the waste at restaurant level W_RTE =_ W_C*_ C_RTE._

Once discarded, food waste could have different destinations, including recovery processes (production of biofuel, re-cycling, composting, etc.), feeding animals (mostly swine) or disposed in landfills. The first option does not involve contact with pigs, and consequently no further studies were performed on it. The model assessed two potential pathways of contact with susceptible swine populations: P_SF_, the probability of domestic pig getting in contact with infected products through swill feeding, and P_LF_, the probability of feral pigs getting in contact with infected products having access to landfills.

The frequency of *swill feeding practices* and the regulations applied, strongly differs depending on the origin of the food waste (i.e. retail and restaurants vs. household food), therefore the household waste was considered separately from the others. For the probability of swill feeding originated from retail (SF_R_) and restaurants (SF_RTE_) we considered the proportion of food waste later used as swill feeding from the retail sector (SF|W_R_) [41] and restaurants (SF|W_RTE_) [41, 42], the authorization of this practice per state (GF), the efficacy of the treatment (Ef) and potential illegal practices (Pil).The retail and restaurants food waste is only permitted to be used as feed for animals in certain states of the US under the regulations of the garbage feeder law [43]. Based on that legislation, food waste should be treated at 212°F for at least 30 minutes, ensuring the inactivation of pathogens. A certain degree of uncertainty about the efficacy of this process (Ef) was included for the states where this practice is allowed (called GF) with a beta distribution assuming that with 95% confidence the heating treatment of the food waste is effective more than 90% of the cases, and most likely 95% efficient. In the states where this practice is forbidden (NGF), certain degree of uncertainty due to illegal practices was assumed (P_il_), by a beta distribution with a 95% of confidence that the swill feeding illegal practices are less common than 10% and most likely under 5%. These assumptions were used to compute beta distributions by the use of BetaBuster [37]. The probability of domestic pigs having access to pork waste from retail by swill feeding per state was estimated as following: SF_R_ = W_R*_ SF|W_R_*(GF*((1- Ef) + P_il_)). For the swill feeding from restaurants (SF_RTE_) the equation is exactly the same but applying the values related with restaurants waste (i.e. W_RTE_ and SF|W_RTE_).

To estimate the probability of swill feeding practices in the households (SF_H_) we employed a similar approach than the one used by [44]. Firstly, we considered the proportion of food waste in households (WH), and the probability of this waste used as swill feeding (SF|W_H_) [45]. Then, the probability of the swine products being consumed in a household with presence of pigs (P_HP_) was estimated considering the number of household with pigs vs the total number of occupied houses in the US [23]. Previous surveys done in the US revealed that the probability of swill feeding practices differ depending on the size of the pig farm [46]. Therefore, considering the proportion of different size’ pig farms per state [47] and the probability of swill feeding practice per size of farm [48], a prorated swill feeding probability was estimated per state (SF|P_HP_). Finally, the risk associated with swill feeding practices with waste food from households was estimated as: SF_H_ = W_C_*(1-C_RTE_)* SF|WH* P_HP_*(SF|P_HP_).

The second potential destination of the food waste that could lead into ASF/ CSF outbreak is *the disposal in landfills* and the potential access of feral pigs to the landfills (P_LF_). As the proportion of free-ranging domestic pigs (without barriers) in the US is very low, the possibility of domestic pig population having access to landfills was not considered in the assessment. To evaluate that risk, firstly we obtained the proportion of food waste discarded in landfills from the retail sector (LF_R_), restaurants (LF_RTE_) [41, 42] and households (95.2%) [45]. After that, a spatial analysis was performed using ArcGIS 10.3 (ESRI ®) to estimate the proportion of landfills per state with potential presence of feral pigs (LF_FP_). For doing that, the maps of feral pigs distribution [49] and information on landfills distribution [50] were overlapped and statistics were obtained per state including the minimum, mean and maximum proportions of the number of landfills, surface and capacity of landfills present in areas with presence of feral pigs vs the total landfills per state. Those values were used to parameterize the probability of feral pigs presence in landfills per state (LF_FP_). No data was found related with the probability of feral pigs having access to the landfills content. Therefore, we assumed a conservative approach, with a minimum probability of feral pigs having access to landfills of 0.05, a most likely probability of 0.1 and maximum of 0.2 (FP_A_). The probability of feral pigs getting in contact with food waste disposed in landfills coming from any of the three sectors was estimated as following:

$$\mathrm{PLF}=\left(W_{R}*LF_{R}+R_{RTE}*LF_{RTE}+W_{H}*LF_{H}\right)*FP_{A*}LF_{FP}$$

Assuming that both exposure routes are mutually exclusive, meaning that they can’t not occur simultaneously (i.e. if not consumed product is used as swill feeding can’t end in a landfill, and vice versa), the final exposure was calculated as the sum of all three probabilities of domestic pigs being exposed by swill feeding (∑*SFi*) plus the probability of feral pigs getting in contact with infected imported swine products disposed in landfills (PLF), all of them calculated at state level.

The results of the models were presented as annual means (95% CI). The annual mean (sum of monthly probabilities) probabilities of ASF /CSF introduction were mapped in ArcGis 10.0 (ESRI®) using Natural Breaks (5 classes) as classification methods calculated by Jenks algorithm [59] and the Cartographic Boundary Shapefiles from the US Census Bureau.

### Combined probabilities

In order to provide a global picture of the risk for the introduction of these diseases in the US, we combined the four analyzed pathways. Firstly, we calculated the annual probabilities of introduction per country for each disease by any of the two pathways analyzed. Considering both pathways (legal import of pigs and legal importation of pig products) as independent events not mutually exclusive, the combined probability per disease was estimated as follows:

$$P_{ASF}=P_{ASFL+}P_{ASFp-}(P_{ASFL*}P_{ASFP})$$

where P_ASF_ is the combined probability of ASF being introduced into the US by legal imports, P_ASFL_ is the probability of ASF being introduced into the US by legal imports of live pigs and P_ASFP_ the probability of ASF being introduced into the US by legal imports of pig products. The same calculations applied for the combined probability of CSF (P_CSF_).

Finally, assuming the introduction of each disease being independent and not mutually exclusive from each other, the final combined probability of introduction of ASF or CSF into the US by legal imports (P_C_) was estimated as follows:

$$P_{C}=(P_{\mathrm{ASF}}\cup P_{CSF})=P_{ASF+}P_{CSF-}(P_{ASF*}P_{CSF})$$

### Sensitivity analysis

Sensitivity analyses were performed for all the models previously described in two steps. Firstly, the most influential parameters in each of the models were identified by calculating the regression coefficients (β_i_) between each input and the annual probability of ASF/CSF introduction in the US. Afterwards, the inputs that were most likely to influence the final results (β_i_ ≥ 0.1) were analyzed in detail using the advanced sensitivity analysis tool of @RISK 5.5 running 500 iterations for each scenario. A total of 10 scenarios were assessed for each selected parameter, by changing the base values in ten consecutive steps, from a minimum of 50% reduction to a maximum of 50% increase.

## Results

### Probability of ASF/CSF introduction into the US by legal imports of live pigs

Considering the current situation of both diseases (as of November 2016), the probability of ASF introduction into the US by legal imports of live pigs was estimated as 3.6*10^−3^ (2.0*10^−4^, 1.5*10^−2^), while the probability of CSF by the same pathway was 2.5*10^−3^ (4.4*10^−5^, 1.1*10^−2^). These values approximately correspond with one outbreak of ASF in 276 years, and one introduction of CSF in 201 years, if conditions remain constant. The overall mean annual probabilities of ASFV being released by imported pigs was 8.4*10^−2^ (6.8*10^−3^, 3.2*10^−1^), whereas for CSFV this probability reached 1.2*10^−1^ (2.1*10^−3^, 5.1*10^−1^). The mean exposure of US domestic pig population to imported pigs (potentially infected either with ASFV or CSFV) pigs was 4.4*10^−2^ (1.6*10^−2^, 8.7*10^−2^).

**Fig 3:**
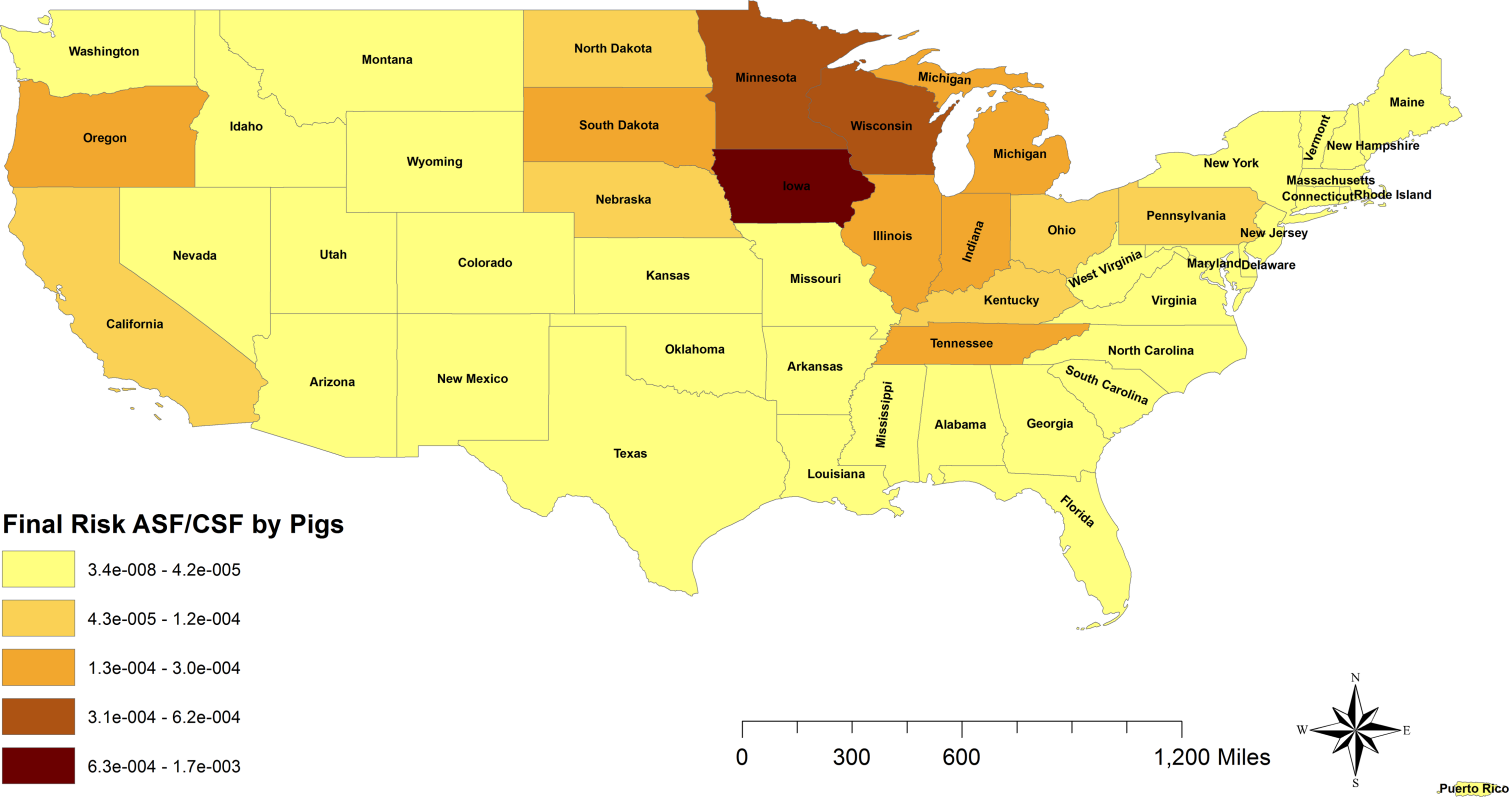
Final risk of ASF/ CSF introduction by legal imports of live swine into the US. The graduated color map represents the final risk (release*exposure) from the highest (darker) to the lowest (lighter).

The distribution of the risk of introductions through imports of live pigs into the US was very similar for both diseases analyzed. Although small differences were found between states for ASF and CSF, when the values were categorized for producing the risk maps, the result was the same, so only one map was presented (Fig 3). The highest probabilities were located in Iowa, Minnesota and Wisconsin, which concentrate 57% of the total probability of both viruses introduction. With 99% of the domestic pigs imported to the US coming from Canada, this was the country of origin that poses the highest risk for both, the introduction of ASF and the introduction of CSF through legal imports of pigs, with annual probabilities of 3.6*10^−3^ for ASF, and 4.9*10^−3^ for CSF. The monthly disaggregation of the risk revealed that January and March were the months at higher risk in both models, including also February for CSF. However, no important seasonal differences were observed for none of the diseases (data not shown).

### Probability of ASF/CSF introduction into the US by legal imports of swine products

**Fig 4:**
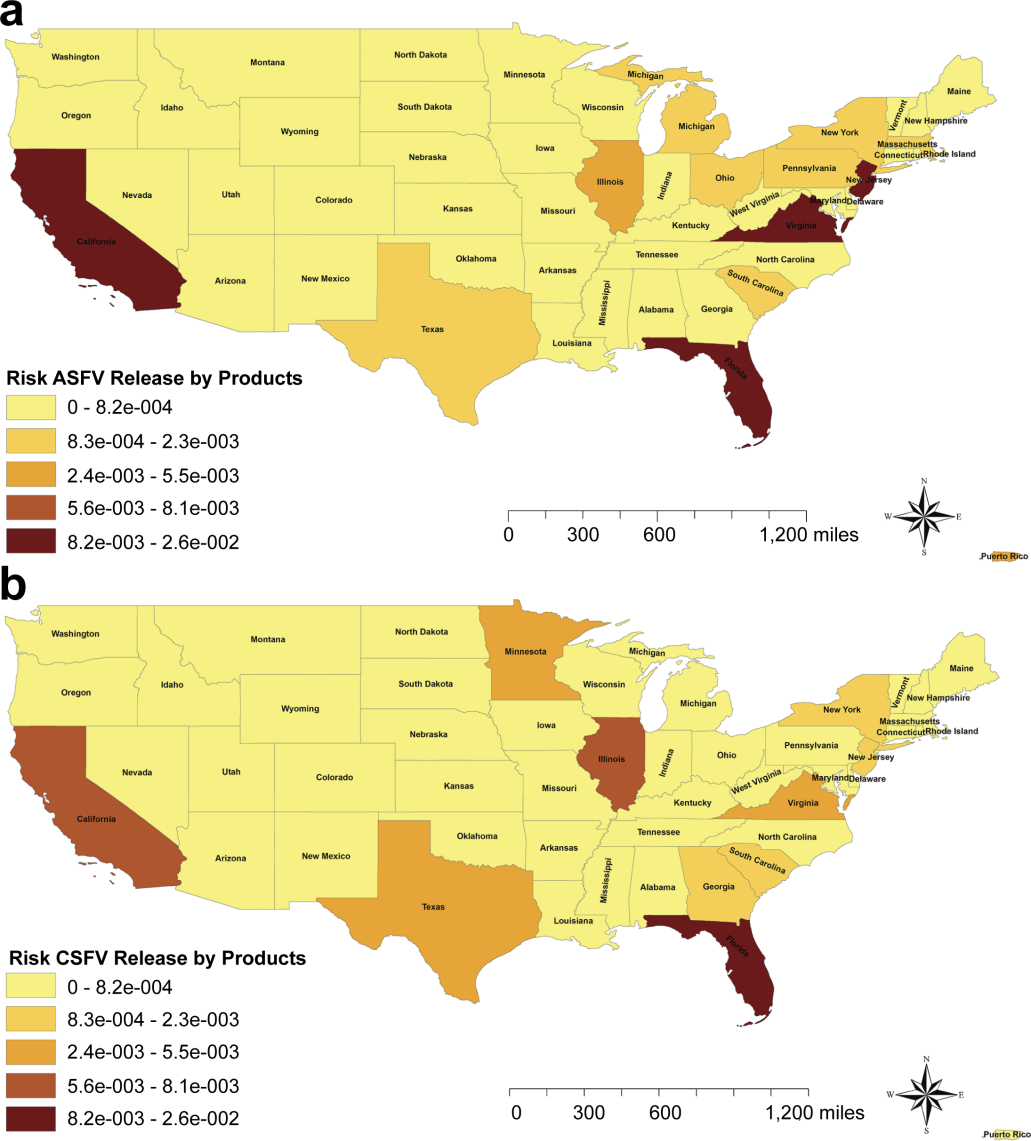
Risk maps of ASFv and CSFv release by legal imports of swine products. (A) Risk of ASFv release into the US. (B) Risk of CSFv release in to the US. The graduated color maps represent the risk from the highest (darker) to the lowest (lighter).

The probabilities of both viruses ASFV/CSFV being released into the US by infected swine products were estimated as 7.8*10^−2^ (8.3*10^−3^, 2.9*10^−1^), and 6.9*10^−2^ (3.9*10^−3^, 2.8*10^−1^), respectively. The location of the risks of ASFV /CSFV being released into the US through import of swine products varies between both viruses and from the risk associated with live pigs imports. Specifically, the risk of ASFV release was highest in the states of New Jersey (2.6*10^−2^), Virginia (1.2*10^−2^), California (9.5*10^−3^) and Florida (9.4*10^−3^). However, the risk of release of CSF potentially infected products was concentrated in Florida (2.6*10^−2^), Illinois (8.1*10^−3^) and California (6.9*10^−3^) (Fig 4). As it can be observed in the map, Florida and California presented relatively high risk for both diseases, either ASF/CSF release.

A total of 91% of the risk of ASFV being released into the US was originated from Denmark (5.8*10^−2^) and Poland (1.4*10^−2^). However, for the introduction of CSF the origins of the risk were wider distributed, being Finland (4.8*10^−2^), Canada (7.1*10^−3^), Cayman Islands (7.1*10^−3^) and Denmark (1.7*10^−3^) the origins of the highest risk.

**Fig 5:**
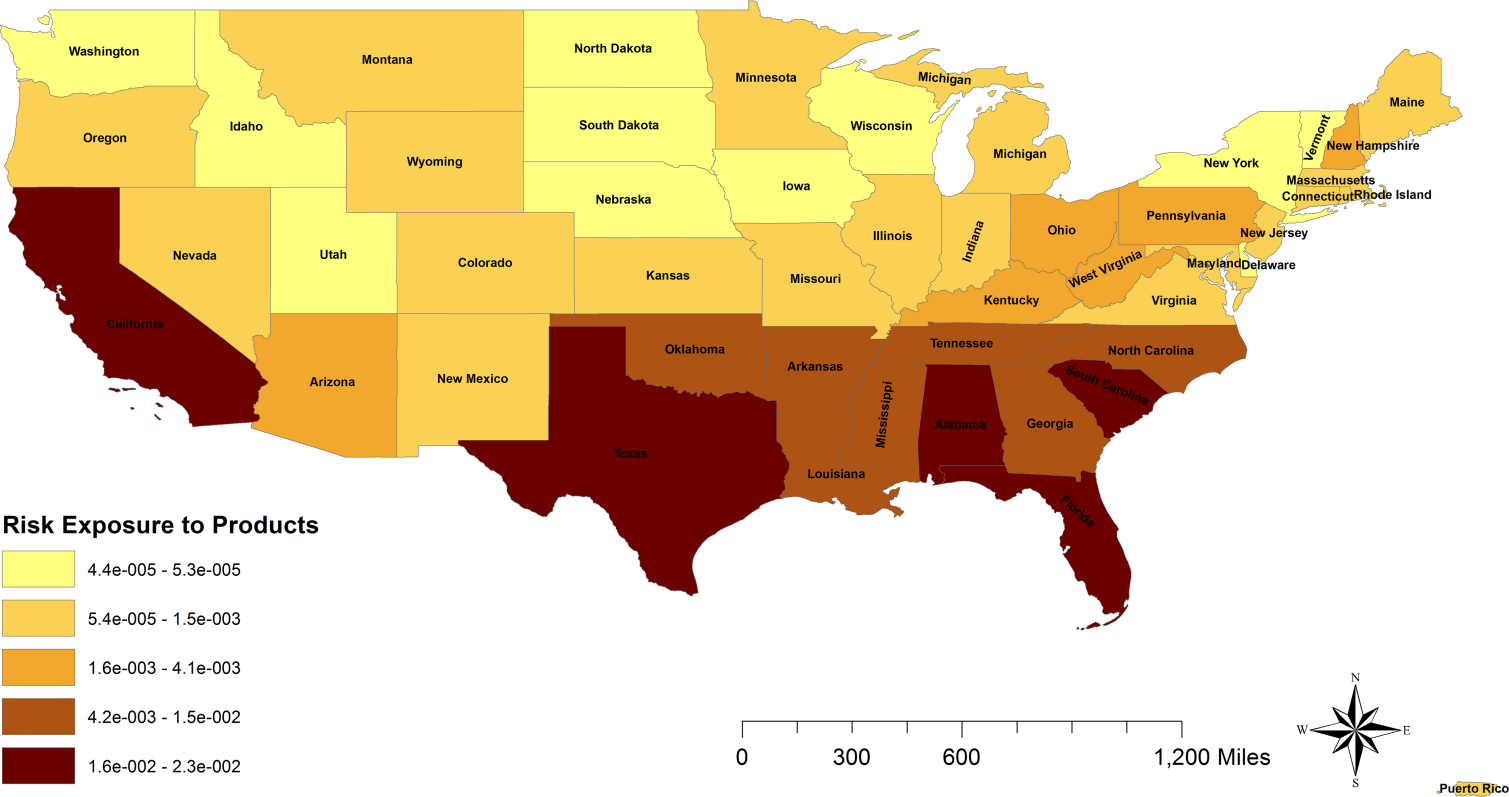
Risk of exposure to legal imports of swine products. The graduated color map represents the risk from the highest (darker) to the lowest (lighter) of US susceptible swine populations being exposed to the legally imported swine products.

The average probability of US swine populations of being exposed to imported products per state was 4.4*10^−3^ (4.6*10^−5^, 1.9*10^−2^), existing important differences between states. This risk of exposure was concentrated in the southern states, including California, mainly due to the presence of feral pigs with potential access to landfills, as well as to the smaller size of swine premises, which present higher risk of using swill feeding (Fig 5). The risk of exposure was almost 10 times higher for the potential access of feral pigs to landfills (2.5*10^−1^) than the risk associated with swill feeding activities to domestic pigs (2.0*10^−2^). The waste originated from households contributed with 64% of the total risk of exposure, mainly due to the disposal of 94.5% of the generated waste in landfills. This risk was followed by the waste from the risk associated with retail sector (20%) and finally the restaurants waste (16%).

**Fig 6:**
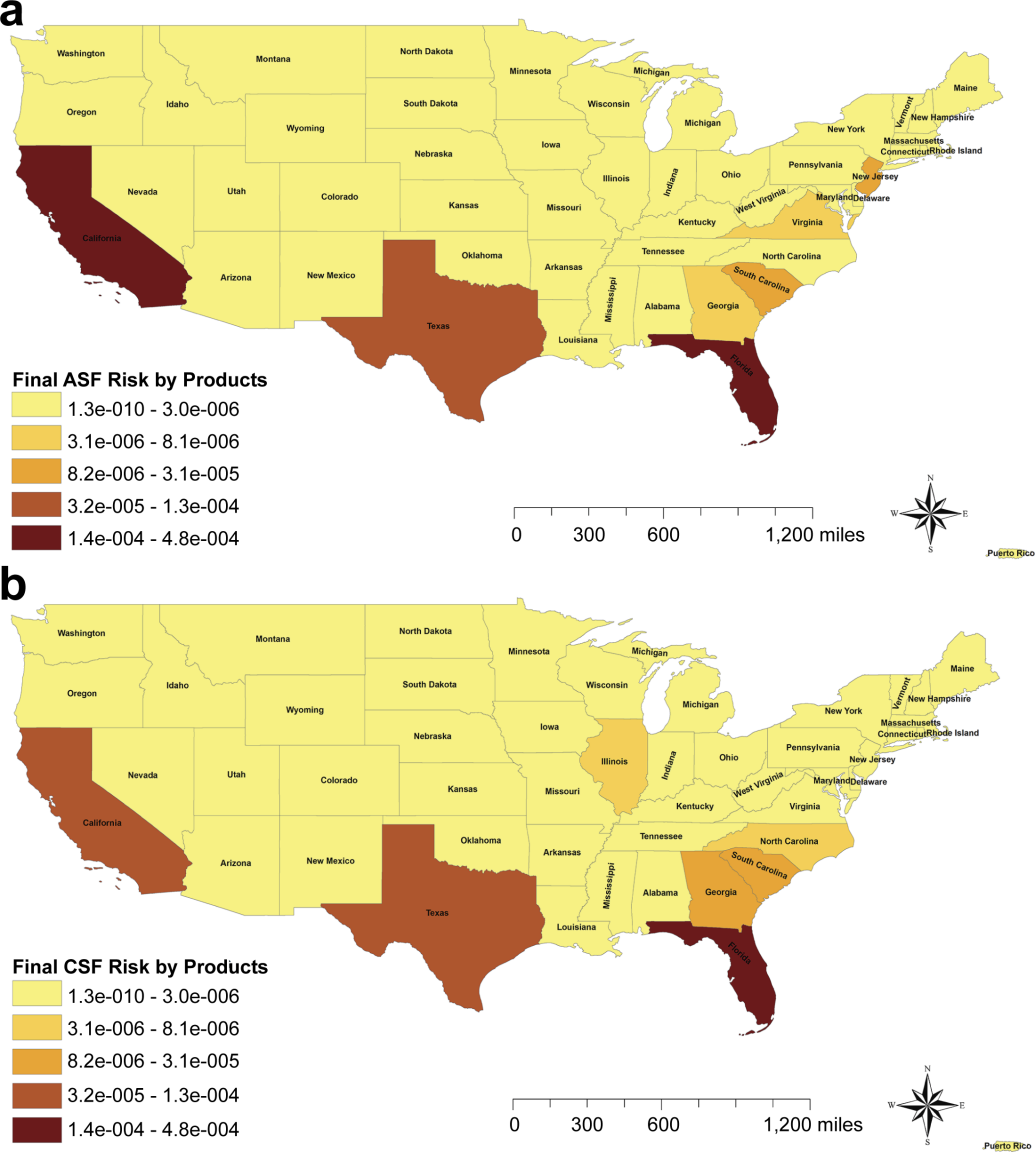
Final risk of ASF and CSF introduction by legal imports of swine products. The graduated color maps represent the final risk (release *exposure) from the highest (darker) to the lowest (lighter). (A) Risk of ASF introduction into the US by legal imports of swine products. (B) Risk of CSF introduction into the US by legal imports of swine products.

Although the risk of release was higher for ASF, the final probability of CSF being introduced into the US through infected swine products was almost two times higher than the risk of ASF by this pathway. Specifically, for ASF the final probability was estimated as 4.5*10^−4^ (4.2*10^−5^, 1.9*10^−3^), and 8.3*10^−4^ (3.8*10^−5^, 3.5*10^−3^) for CSF. Those probabilities were approximately eight times (ASF) and 6 times (CSF) lower than the estimated probabilities of introduction through imports of live pigs. The final risk maps for both diseases were quite similar, being in both cases Florida and California the states at highest risk (Fig 6). However, while 90% of the total risk of CSF was concentrated in these states, the risk of ASF was broader distributed. In contrast to the live pig pathway, the ASF/CSF risk associated with the imports of swine products presents certain seasonality being April and October the months at highest risk for both diseases analyzed.

### Combined probabilities

The combined probability of ASF introduction into the US through legal importations (of pigs or products) was estimated as 2.2*10^−3^(1.6*10^−4^, 8.7*10^−3^). For CSF, the combined probability resulted in 3.3*10^−3^(8.1*10^−5^, 1.5*10^−2^). The final combined probability, which estimates the risk of ASF or CSF being introduced into the US by legal importations of animals or products was estimated as 5.5*10^−3^(2.4*10^−4^, 2.3*10^−2^), which is approximately equivalent to one outbreak every 181 years.

### Sensitivity analysis

**Fig 7:**
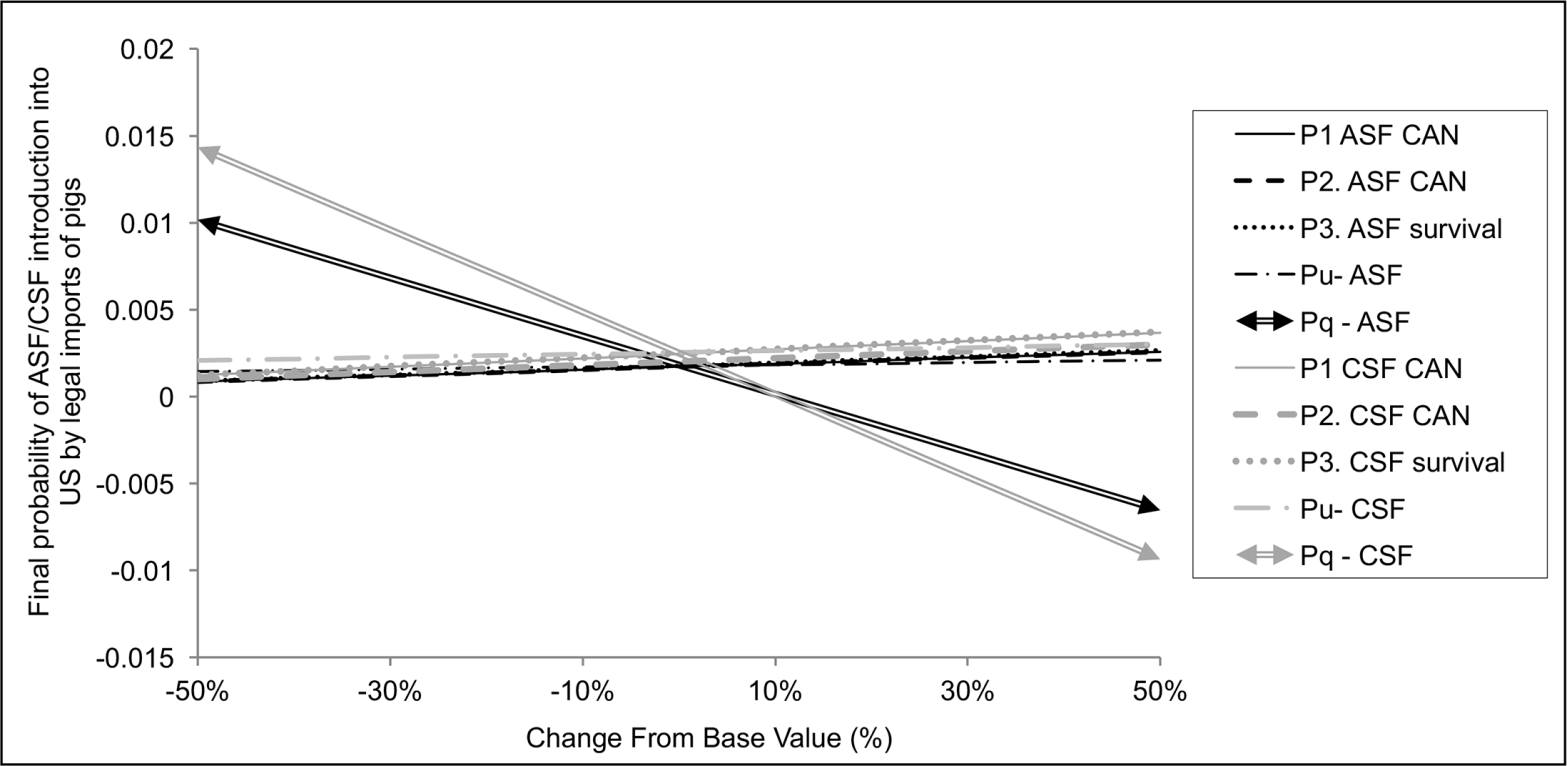
Advanced sensitivity analysis for the models of ASF/CSF risk of introduction by pigs imports. The spider graph plots the percent change of the selected input parameters against the output results.

Based on the correlation coefficient assessment (β_i_ ≥ 0.1), the following inputs were selected for the advanced sensitivity analysis from the pigs imports model: the probability of ASF/CSF infection in Canada (P1 CAN), the probability of selecting an infected pig from Canada (P2 CAN), the probability of survival to ASF/CSF infection (P3), the probability of pigs being undetected during quarantine (P_u_) and the probability of imported pigs going through quarantine (P_q_). The advanced sensitivity analysis reveals that both models were only noticeable influenced by one parameter: Pq or the probability of imported pigs going through quarantine (Fig 7). The rest of the parameters analyzed didn’t influence substantially the final result, even when they were increased or decreased up to 50%.

**Fig 8:**
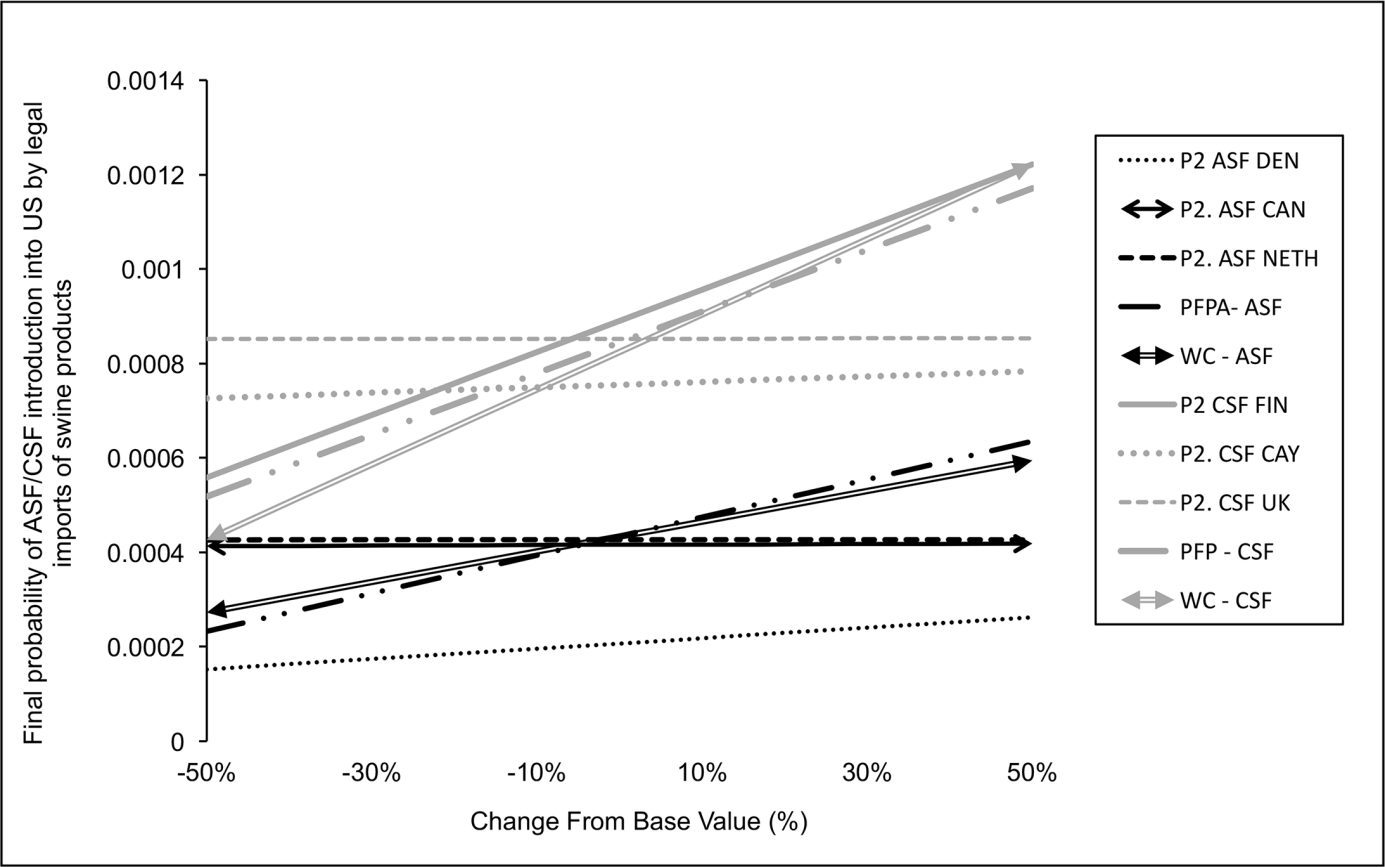
Advanced sensitivity analysis for the models of ASF/CSF risk of introduction by swine products imports. The spider graph plots the percent change of the selected input parameters against the output results.

Five parameters were selected from each legal imports of products model for the advanced sensitivity analysis based on their correlation coefficients. The probability of feral pigs accessing to landfills (FP_A_) and the food losses at consumer level (W_C_) were selected for both models. In addition, from the ASF model of swine products the probability of selecting infected meat from Denmark (P2 ASF DEN), Canada (P2 ASF CAN) and The Netherlands (P2 ASF NETH) were included. Whereas for the CSF model, the probability of selecting infected meat from Finland (P2 CSF FIN), Cayman Island (P2 CSF CAY) and United Kingdom (P2 CSF UK) were selected (β_i_ ≥ 0.1). Based on the advanced sensitivity analysis, both models were highly influenced by the likelihood of feral pigs accessing to landfills and the food losses at consumer level (Fig 8). Based on the scenarios run on the ASF model, the rest of the parameters assayed have none (P2 ASF CAN and NETH) or very little influence (P2 ASF DEN). In contrast, the probability of selecting pigs from Finland (P2 CSF FIN) highly influenced the CSF model results.

## Discussion

Following the OIE guidelines [20], four quantitative risk assessment models were developed to evaluate the risks of ASF and CSF introduction into the US by legal importations of live pigs and products during the high risk period (i.e., time between infection and detection/notification in the source country). The primary aim of import risk analysis is to provide importing countries an objective and defensible method for assessing the risks associated with the importation of animals and animal related products (i.e. genetic material, feedstuffs, biological products and pathological material) [60], as well as to identify hazards and how those hazards could eventually become a risk. Therefore, risk analysis should be always transparent, based on the best available information and fully referenced, as their results can be used to regulate international trade. In this study, when available, only accredited sources of data were used. For those parameters for which no detailed information exists, expert opinion and certain assumptions (assuming the highest risk scenario) were included in the model, being all of them fully described in detail in Tables 2, 3 and 4, ensuring the transparency of the assessment. The intense sensitivity analyses performed in the study allowed us to estimate the impact of these parameters in the final model outcomes, and their relative importance in the final results.

In the present analysis, significant novelties were incorporated to address some of the biggest challenges previously identified in similar studies [61]. For example, the estimation of the risk of infection in the origin countries has been always a critical parameter, difficult to address in the import risk assessments. If the disease is present in the origin countries, the prevalence data is normally used as proxy for the risk of infection in origin [20]. However, when the disease is absent in the countries of origin, estimations based on the time from the last outbreak are sometimes used [61–63], assuming that the occurrence of the diseases is seasonal (i.e. disease outbreaks will occur every X years). This assumption could be valid for some vector-borne diseases highly dependent on climatic factors (i.e. Rift Valley Fever). However, it is not valid for the two diseases analyzed here (ASF and CSF), as the outbreaks in new affected areas are commonly initiated by movement of infected animals or contaminated material.

Therefore, in this study we used a Susceptible-Infected compartmental model, assuming that the risk of a country to become infected by ASF/CSF strongly depends on their commercial relationships with currently infected countries. We are aware that this approach only addresses one of the routes of introduction of animal diseases in free countries, and other important sources of infection were not considered (i.e. movement of wild animals, illegal imports, etc.). However, the lack of detailed data and controversy associated with the estimation of those other pathways make these estimations very difficult and require to be supported by a series of risk assessments of each of the source countries, which was out of the scope and possibilities of this study. Therefore, although is not the perfect solution, it was considered that this approach provides more realistic information than traditional estimates, and could be easily applied in other risk assessment models.

Another strength of this study was the integration of the release and exposure assessments in one single stochastic model for the assessment of the risk associated with imported products. The integration of both steps in a common model is not common, as in most of the cases, the quality of information are not comparable and each step is assessed separately (i.e. release and exposure in [64]). In addition, this is the first time that the risk of exposure of US swine populations through swill feeding and landfill disposal are numerically compared. Around one third of the food produced in the world for human consumption (approximately 1.3 billion tons) gets lost or wasted every year [65]. Innovative approaches for promoting food waste reduction are necessary, as well as new options to ensure the correct disposal of food waste, maximizing the profitability of proteins. One of the options for that is swill feeding (which is mostly used to feed swine). Although the practice is controversial as it could involve serious health risks, as demonstrated with the introduction of ASF in Georgia in 2007 [7] or the spread of many other TADs, swill feeding has been historically largely used as it is a way to maximize the use of the food.

If done in controlled manner (with appropriate heat treatment, regulations and controls in place), our model suggests that it implies less risks than disposing the food waste in landfills.

In our model, the risk associated with swill feeding practices was mostly related with the food waste generated in households (64% of the risk of swill feeding was associated with waste from households vs retail and restaurants). Opposite to EU where swill feeding is totally banned, many states in the US allow swill feeding to swine under certain conditions (properly heat-treated and fed by a licensed facility) [66]Therefore, in order to reduce the risk of exposure and optimize the use of meat, swill feeding practices should be promoted in a controlled manner and communication and informative campaigns should be performed in small swine premises or swine hobby farmers.

Most of the food waste generated in our houses ends in landfills (94.5%) [42], and many of those landfills are located in areas are inhabited by feral pigs, that potentially have access and get in contact with the material discarded on them. Our results indicate that the potential risk of exposure of feral pigs to food waste present in open landfills is not negligible (2.5*10^−2^), posing a higher risk compared to other exposure pathways analyzed here as the swill feeding activities in domestic pigs. In addition, although not included in this study, feral pig could potentially have access to the food waste in bins and containers, before it arrives to the landfills, as well as contacting domestic pigs. The contacts and potential transmission of pathogens between domestic pigs and feral pigs has been documented in certain areas of the US [67] and pose a risk for disease controlling. In these models this pathway of exposure was not included, due to the characteristics of swine pigs imported into the US (99.9% are feeder pigs coming from Canada [38] with destination to large feeding units with no outdoor access). However, it would be an interesting research area to explore for other scenarios. The sensitivity analysis revealed that the probability of feral pigs accessing landfills (which was a high risk scenario assumption) and the proportion of food waste at consumer level highly influenced our results. Therefore, we strongly recommend further research efforts to evaluate the potential access of feral pigs to landfills and other sources of food waste, and estimate the consequences derived from it, as this could be a risk not only for ASF and CSF but for many other diseases/ health issues that could be transmitted through food waste (i.e. E.coli, antimicrobial resistance, toxins, etc.).

Despite the global burden situation of ASF and CSF, and the assumptions used in the model, the risk of introduction of either ASF or CSF into the US by legal import of animal and products is considerably low (combined probability of 5.5*10^−3^). However, it is important to remember that other pathways should be analyzed such as the importation of other biological products (semen, ova, etc.), animal feed and, importantly, illegal pathways, including the waste from planes and other international transports, in order to have a more complete picture of the risk of ASF and CSF introduction into the US. Among the four pathways analyzed, the imports of pigs posed the highest risk for the introduction of CSF into the US. This could be caused, firstly, due to the fact that CSF is more widely distributed in the world, and the probabilities of CSF infection in origin derived from the SI model were higher comparing with ASF. Secondly, the risk associated with the legal import of swine products was lower in both diseases (4.5*10^−4^ for ASF and 3.8*10^−4^ for CSF) compared to the live swine imports (3.6*10^−3^ for ASF and 4.9*10^−3^ for CSF), potentially due to the lower risk of exposure to the imported products, as they usually go directly to human consumption. However, the risk of ASF and CSF released by imports of animal products was considerable higher (0.1), which reflects the importance of controlling the exposure to these products to avoid potential outbreaks of these diseases in the US.

As it was expected, the risk of ASF and CSF introduction by legal imports of pigs was concentrated in the US pork production states, which are the main importers of pigs in the country as Iowa (35%), Minnesota (12%) and Wisconsin (10%). The models presented here estimated the existing risk associated with exporting animals before the detection in the country of origin (high risk period), assuming that US will not accept importation of swine from ASF or CSF infected countries. Canada is an ASF/CSF free country but the risk rises with increasing the volume of commodity imported. According to FAOSTAT [24], in the past Canada has had trade of live pigs with non-free countries such as Russia (ASF/CSF), Colombia (CSF), Republic of Korea (CSF), China (CSF), Poland (ASF), Peru (CSF), Italy (ASF) and Philippines (CSF). The strong connections between both markets implies that the swine disease situation in Canada is crucial to the US swine market, for ASF and CSF and any other infectious disease potentially appearing. Consequently, as soon as any change occur in swine status in Canada, the US should re-evaluate its risk levels, and consider the implementation of preventive measures.

The probability of domestic pigs in the US being exposed to a potentially infected (either with ASFV or CSFV) imported swine was estimated as 4.35*10^−2^. However, the results of the sensitivity analysis identified the probability of imported pigs going through quarantine (Pq) as the essential parameter in the model. Therefore, the correct application of quarantine procedures is an essential component for maintaining the free status and reduce the risks associated with animal importations.

For the legal importation of live pigs, no differences were found on the location of the risk between the analyzed diseases (ASF or CSF). However, for the risk associated with the legal imports of swine products, the location of the risk substantially varies between ASF and CSF, due to the trade differences between the states. Whereas for ASF the probability of release was highest in New Jersey (33%), Florida (14%), California (13%) and Virginia (11%); for CSF the risk of release was concentrated in Florida (38%), Illinois (12%), California (10%), and Texas (8%) (Fig 5). The probability of exposure to swine products was concentrated in the southern states and California (Fig 6) due to the abundance of feral pigs that could access to landfills and usually smaller pig farms that present higher probability of using swill feeding [47, 48]. As a result of the combination of release and exposure risks, the final risk of ASF/CSF being introduced into the US through legal products importations is highly concentrated in the states of Florida (38% for ASF and 58% of CSF risk), California (39% of ASF risk and 16% of CSF) and Texas (11% of ASF risk vs 16% CSF risk). In this pathway, although Canada was again the highest risk country for the origin of these products, other countries presented also a relative high importance in this pathway including Denmark and Poland for ASF. The imports from Poland only represents 3.4% of the total products imported. However, it was the second highest risk origin country due to the presence of ASF in the country since February 2014. Interestingly, the imports of swine products from Poland have not been stopped for ASF, but continue gradually increasing. In this case of an already infected country, the preventive measures should be focused on analyzing and ensuring the freedom from disease in the units approved for exports. Additional checks of biosecurity compliance in origin farms, regionalization procedures, or even periodic diagnostic testing of the swine products in origin would be also recommended. In the case of CSF introduction, surprisingly Finland was one of the highest risk, potentially due to the swine trade connections with China, Russia, Estonia and Poland [24]. Indeed, the probability of selecting infected swine products from Finland was one of the most influential parameters of the model for CSF risk of imported products (Fig 8).

Although the consequences of the introduction of ASF and CSF into the US were not considered in this work, the example of previous outbreaks in other free territories suggest a potential huge economic impact. Presumable, the impact would be more serious in the case of ASFV introduction, as no vaccines are available for its control. However, in the case of CSF, there are highly efficacious and safe vaccines available, including a bait format for oral immunization which could be used in wild boar and even in domestic pigs in backyard conditions [17].

Quantitative risk assessments present certain benefits and advantages versus qualitative models, as they incorporate the variability of data, uncertainty of the estimations and evaluate the influence of the parameters in the final outcome through sensitivity analysis. However, this type of analysis results in very time intensive process due to the substantial efforts for collecting all the data required plus the computational requirements. For example, in this study, more than 15,000 inputs were used in the models for the legal importations of pig products. On the other hand, once the models’ structures are defined, data sources identified and available, the models can be easily updated. The studies developed here not only served to estimate the probabilities of ASF and CSF introduction through legal imports, but will constitute the basis for the documentation and quantitative analysis of the risk of other FADs into the US, that would help to prevent the negative consequences associated with these types of diseases. The epidemiological information obtained in the present study could be used to develop risk-based surveillance, prevention and early detection strategies that would help to prevent ASF/CSF introduction and protect US swine livestock and consumers as well as allocate resources effectively and efficiently. In addition, the model parameters and calculations of the present study will be integrated in an online user-friendly risk assessment platform (Disease BioPortal™ accessible from http://bioportal.ucdavis.edu/) for the easy update and visualization of the risk estimates. Other pathways of introduction such as the illegal importation of pork and other swine products into the US by the different ports of entry (airports, maritime, mail, etc.) are currently being addressed and will be also incorporated in the platform.

## Conclusion

Four quantitative stochastic risk assessments models were developed to estimate the risk of ASF/CSF introduction into the US through legal importation of live swine and swine products during the high risk period. The models’ results suggest that the risk of both diseases being introduced into the US through the analyzed pathways was very low, being the risk of CSF by legal imports of pigs the analyzed pathway that poses the highest risk. The risk of introduction through live swine imports is higher for CSF than ASF, being for both diseases concentrated in the pork production states (Iowa, Minnesota and Wisconsin), with most of the live pigs coming from Canada. In contrast, the final risk of ASF/CSF introduction for the products model was concentrated in states of California, Florida and Texas. However, the risk of entrance of potentially infected swine products into the US clearly differs between CSF and ASF. The epidemiological information obtained in the present study could be useful to develop prevention and early detection strategies that would help to prevent ASF/CSF introduction as well as allocate resources effectively and efficiently.

## References

[1] OIE. World Organisation for Animal Health 2016. Available from: http://www.oie.int/animal-health-in-the-world/oie-listed-diseases-2016/.

[2] Sánchez-Vizcaíno JM, Arias M. African swine fever In: Zimmerman J, Karriker LA, Ramirez A, Schwartz K, Stevenson G, editors. Diseases of swine. 10 ed Ames, Iowa: Blackwell Publishing Professional; 2012 p. 396–404.

[3] ICTV. The International Committee on Taxonomy of Viruses 2016. Available from: http://www.ictvonline.org/virusTaxonomy.asp.

[4] Plowright W, Parker J, Pierce MA. African Swine fever virus in ticks (Ornithodoros moubata) collected from animal burrows in Tanzania. Nature. 1969;221:1071–3. 5813153 10.1038/2211071a0

[5] Costard S, Mur L, Lubroth J, Sanchez-Vizcaino JM, Pfeiffer DU. Epidemiology of African swine fever virus. Virus Res. 2013;173(1):191–7. doi: 10.1016/j.virusres.2012.10.030 . 23123296 10.1016/j.virusres.2012.10.030

[6] Montgomery RE. On a form of swine fever occurring in British East Africa. J Comp Pathol. 1921;34:59–191.

[7] Rowlands RJ, Michaud V, Heath L, Hutchings G, Oura C, Vosloo W, et al African swine fever virus isolate, Georgia, 2007. Emerging infectious diseases. 2008;14(12):1870–4. Epub 2008/12/03. doi: 10.3201/eid1412.080591 ; PubMed Central PMCID: PMCPMC2634662. 19046509 10.3201/eid1412.080591 PMC2634662

[8] Gogin A, Gerasimov V, Malogolovkin A, Kolbasov D. African swine fever in the North Caucasus region and the Russian Federation in years 2007–2012. Virus research. 2013;173(1):198–203. doi: 10.1016/j.virusres.2012.12.007 . 23266725 10.1016/j.virusres.2012.12.007

[9] Sanchez-Vizcaino JM, Mur L, Gomez-Villamandos JC, Carrasco L. An update on the epidemiology and pathology of African swine fever. Journal of comparative pathology. 2015;152(1):9–21. Epub 2014/12/03. doi: 10.1016/j.jcpa.2014.09.003 . 25443146 10.1016/j.jcpa.2014.09.003

[10] Sánchez-Vizcaíno J, Mur L, Sánchez-Matamoros A, Martínez-López B. African Swine Fever: new challenges and measures to prevent its spread 2014 Available from: www.oie.int.

[11] Saatkamp HW, Berentsen PB, Horst HS. Economic aspects of the control of classical swine fever outbreaks in the European Union. Veterinary microbiology. 2000;73(2–3):221–37. Epub 2000/04/28. . 10785330 10.1016/s0378-1135(00)00147-4

[12] Moennig V, Floegel-Niesmann G, Greiser-Wilke I. Clinical signs and epidemiology of classical swine fever: a review of new knowledge. Veterinary journal. 2003;165(1):11–20. . 12618065 10.1016/s1090-0233(02)00112-0

[13] Luo Y, Li S, Sun Y, Qiu HJ. Classical swine fever in China: a minireview. Veterinary microbiology. 2014;172(1–2):1–6. Epub 2014/05/06. doi: 10.1016/j.vetmic.2014.04.004 . 24793098 10.1016/j.vetmic.2014.04.004

[14] OIE WAHIS. OIE WAHIS Interface 2016. Available from: http://www.oie.int/wahis_2/public/wahid.php/Wahidhome/Home.

[15] USDA Economic Research Service. Hogs & Pork 2016 [November 3 2016]. Available from: http://www.ers.usda.gov/topics/animal-products/hogs-pork/.

[16] Madera R, Gong W, Wang L, Burakova Y, Lleellish K, Galliher-Beckley A, et al Pigs immunized with a novel E2 subunit vaccine are protected from subgenotype heterologous classical swine fever virus challenge. BMC veterinary research. 2016;12(1):197 Epub 2016/09/11. doi: 10.1186/s12917-016-0823-4 ; PubMed Central PMCID: PMCPMC5016919. 27612954 10.1186/s12917-016-0823-4 PMC5016919

[17] Rossi S, Staubach C, Blome S, Guberti V, Thulke HH, Vos A, et al Controlling of CSFV in European wild boar using oral vaccination: a review. Front Microbiol. 2015;6:1141 Epub 2015/11/12. doi: 10.3389/fmicb.2015.01141 ; PubMed Central PMCID: PMCPMC4615961. 26557109 10.3389/fmicb.2015.01141 PMC4615961

[18] Costard S, Wieland B, de Glanville W, Jori F, Rowlands R, Vosloo W, et al African swine fever: how can global spread be prevented? Philos Trans R Soc Lond B Biol Sci. 2009;364(1530):2683–96. Epub 2009/08/19. doi: 10.1098/rstb.2009.0098 ; PubMed Central PMCID: PMCPMC2865084. 19687038 10.1098/rstb.2009.0098 PMC2865084

[19] Martinez-Lopez B, Perez AM, Sanchez-Vizcaino JM. A stochastic model to quantify the risk of introduction of classical swine fever virus through import of domestic and wild boars. Epidemiology and infection. 2009;137(10):1505–15. Epub 2009/02/27. doi: 10.1017/S0950268808001623 . 19243649 10.1017/S0950268808001623

[20] OIE World Organisation for Animal Health. Handbook on Import Risk Analysis for Animals and Animal Products Quantitative Risk Assessment. France: OIE; 2004. 126 p.

[21] OIE World Organisation for Animal Health. Handbook on Import Risk Analysis for Animals and Animal Products Introduction and qualitative risk analysis. France: OIE; 2010 88 p. doi: 10.1111/j.1539-6924.2010.01386.x

[22] USDA Global Agricultural Trade System. 2016. Available from: https://apps.fas.usda.gov/gats/default.aspx.

[23] US Census Bureau Trade Online. 2016. Available from: https://usatrade.census.gov/.

[24] FAOSTAT. 2016. Available from: http://faostat.fao.org/.

[25] de Carvalho Ferreira HC, Backer JA, Weesendorp E, Klinkenberg D, Stegeman JA, Loeffen WL. Transmission rate of African swine fever virus under experimental conditions. Veterinary microbiology. 2013;165(3–4):296–304. Epub 2013/05/15. doi: 10.1016/j.vetmic.2013.03.026 . 23664069 10.1016/j.vetmic.2013.03.026

[26] Stegeman JA, Elbers AR, Boum A, de Jong MC. Rate of inter-herd transmission of classical swine fever virus by different types of contact during the 1997–8 epidemic in The Netherlands. Epidemiology and infection. 2002;128(2):285–91. Epub 2002/05/11. ; PubMed Central PMCID: PMCPmc2869822. 12002547 10.1017/s0950268801006483 PMC2869822

[27] R Core Team. R: A language and environment for statistical computing. R Foundation for Statistical Computing, Vienna, Austria 2016.

[28] Csardi G, Nepusz T. The igraph software package for complex network research, InterJournal, Complex Systems 2006. Available from: http://igraph.org.

[29] Butts C. SNA: Tools for Social Network Analysis. R package version 2.4. 2016. Available from: https://CRAN.R-project.org/package=sna.

[30] Martínez-López B, Perez A, Sánchez-Vizcaíno J. A stochastic model to quantify the risk of introduction of classical swine fever virus through import of domestic and wild boars. Epidemiology and infection. 2009;137(10):1505–15. doi: 10.1017/S0950268808001623 19243649 10.1017/S0950268808001623

[31] Spickler AR, Roth JA. Peste porcina africana Iowa State University, College of Veterinary Medicine 2006 Available from: http://www.cfsph.iastate.edu/Factsheets/es/peste_porcina_africana.pdf.

[32] National Agricultural Biosecurity Center. Pathways Analysis of Classical Swine Fever (CSF) risk to the United States 2004. Available from: http://www.k-state.edu/nabc/docs/2004-PA-CSF.pdf.

[33] Murray AC, Johnson CP. Impact of the halothane gene on muscle quality and pre-slaughter deaths in Western Canadian pigs. Can J Anim Sci. 1998;78:543–8.

[34] Animal and Plant Health Inspection Service USDA § 93.503. 1990. Available from: https://www.gpo.gov/fdsys/pkg/CFR-1998-title9-vol1/pdf/CFR-1998-title9-vol1-sec93-503.pdf.

[35] Romero L. Spanish Ministry of Agriculture, MAPA, personal communication.

[36] Eurostat. Pig farming sector—statistical portrait 2014. Available from: http://ec.europa.eu/eurostat/statistics-explained/index.php/Pig_farming_sector_-_statistical_portrait_2014.

[37] Su C-L. BetaBuster. Available from: http://cadms.ucdavis.edu/diagnostictests/betabuster.html.

[38] USDA USDoA-. Hogs & Pork trade 2017. Available from: https://www.ers.usda.gov/topics/animal-products/hogs-pork/trade/#trade.

[39] Agency). USEEP. Food Waste Management in the United States, 2014. 2016.

[40] Office USEEPAR. Summary Analysis of Massachusetts Commercial/Institutional Food Waste Generation Data. 2011.

[41] BSR. Analysis of U.S. Food Waste Among Food Manufacturers, Retailers, and Restaurants. 2014.

[42] United States Environmental Protection Agency. Postconsumer Food Diverted Through Donation, Animal Feed, Anaerobic Digestion, and Composting for 2013. 2015.

[43] USDA APHIS. Swine Health Protection; Feeding of Processed Product to Swine. [Docket No APHIS–2008–0120]. 2009;74(63).

[44] Hernandez-Jover M, Schembri N, Holyoake PK, Toribio JL, Martin PA. A Comparative Assessment of the Risks of Introduction and Spread of Foot-and-Mouth Disease among Different Pig Sectors in Australia. Frontiers in veterinary science. 2016;3:85 Epub 2016/10/08. doi: 10.3389/fvets.2016.00085 27713881 10.3389/fvets.2016.00085 PMC5031773

[45] United States Environmental Protection Agency. Municipal Solid Waste Generation, Recycling, and Disposal in the United States: Facts and Figures for 2012. EPA United States Environmental Protection Agency 2014;EPA-530-F-14-001:13.

[46] United States Environmental Protection Agency. Food waste scoping analysis. 2014.

[47] USDA APHIS NAHMS. Swine 2012 Part I: Baseline Reference of Swine Health and Management in the United States, 2012 2012. Available from: https://www.aphis.usda.gov/animal_health/nahms/swine/downloads/swine2012/Swine2012_dr_PartI.pdf.

[48] USDA APHIS NAHMS. Small-Enterprise Swine 2007. Reference of Management Practices on Small-Enterprise Swine Operations in the United States, 2007 2007. Available from: https://www.aphis.usda.gov/animal_health/nahms/swine/downloads/swine2007/Swine07_dr_SmallSwine.pdf.

[49] USDA. Feral Swine populations 2015 by county. Southeastern Cooperative Wildlife Disease Study 2015. Available from: http://swine.vet.uga.edu/nfsms/information/map2015.htm.

[50] United States Environmental Protection Agency. LMOP Database 2016. Available from: https://www.epa.gov/lmop.

[51] Buzby J, Bentley J, Padera B, Campuzano J, Ammon C. Updated Supermarket Shrink Estimates for Fresh Foods and Their Implications for ERS Loss-Adjusted Food Availability Data. Economic Information Bulletin, 2016.

[52] Buzby J, Wells F, Axtman B, Mickey J. Supermarket Loss Estimates for Fresh Fruit, Vegetables, Meat, Poultry, and Seafood and Their Use in the ERS Loss-Adjusted Food Availability Data. Economic Information Bulletin, 2009.

[53] FAO. Global food losses and food waste–Extent, causes and prevention. 2011.

[54] Buzby J, Wells H, Hyman J. The Estimated Amount, Value, and Calories of Postharvest Food Losses at the Retail and Consumer Levels in the United States. Economic Information Bulletin, 2014.

[55] USDA. Agricultural Research Service. 1994–96 Continuing Survey of Food Intakes by Individuals and 1994–96 Diet and Health Knowledge Survey. 2000.

[56] Davis C, Lin B. Factors Affecting U.S. Pork Consumption. 2005.

[57] USDA APHIS. Rules and Regulations. 2009.

[58] USDA Economic Research Service. ARMS Farm Financial and Crop Production Practices 2016. Available from: http://www.ers.usda.gov/data-products/arms-farm-financial-and-crop-production-practices/tailored-reports-farm-structure-and-finance.aspx#P5c4799ca4f61405682bd0c34bee3c10f_25_66iT0R8T0R0x0.

[59] Environmental Systems Research Institute (ESRI). ArcGIS Release 10.0. Redlands, CA.2010.

[60] Welte V. Risk Analysis and OIE 2016. Available from: http://www.fao.org/docrep/003/x7354e/x7354e12.htm.

[61] Mur L, Martinez-Lopez B, Martinez-Aviles M, Costard S, Wieland B, Pfeiffer DU, et al Quantitative risk assessment for the introduction of African swine fever virus into the European Union by legal import of live pigs. Transboundary and emerging diseases. 2012;59(2):134–44. Epub 2011/08/13. doi: 10.1111/j.1865-1682.2011.01253.x . 21831148 10.1111/j.1865-1682.2011.01253.x

[62] Mur L, Martinez-Lopez B, Costard S, de la Torre A, Jones BA, Martinez M, et al Modular framework to assess the risk of African swine fever virus entry into the European Union. BMC veterinary research. 2014;10:145 Epub 2014/07/06. doi: 10.1186/1746-6148-10-145 ; PubMed Central PMCID: PMCPMC4112856. 24992824 10.1186/1746-6148-10-145 PMC4112856

[63] Sanchez-Vizcaino F, Perez A, Lainez M, Sanchez-Vizcaino JM. A quantitative assessment of the risk for highly pathogenic avian influenza introduction into Spain via legal trade of live poultry. Risk analysis: an official publication of the Society for Risk Analysis. 2010;30(5):798–807. Epub 2010/02/09. doi: 10.1111/j.1539-6924.2009.01351.x . 20136740 10.1111/j.1539-6924.2009.01351.x

[64] Costard S, Jones BA, Martinez-Lopez B, Mur L, de la Torre A, Martinez M, et al Introduction of African swine fever into the European Union through illegal importation of pork and pork products. PloS one. 2013;8(4):e61104 Epub 2013/04/25. doi: 10.1371/journal.pone.0061104 ; PubMed Central PMCID: PMCPmc3627463. 23613795 10.1371/journal.pone.0061104 PMC3627463

[65] FAO. Save food: Global Initiative on Food Loss and Waste Reduction 2016. Available from: http://www.fao.org/save-food/resources/keyfindings/en/.

[66] Broad Leib E, Balkus O, Rice C, Maley M, Taneja R, Cheng R, et al Leftovers for livestock: A Legal Guide for Using Food Scraps as Animal Feed 2016 Available from: http://www.chlpi.org/wp-content/uploads/2013/12/Leftovers-for-Livestock_A-Legal-Guide_August-2016.pdf.

[67] Wyckoff AC, Henke SE, Campbell TA, Hewitt DG, VerCauteren KC. Feral swine contact with domestic swine: a serologic survey and assessment of potential for disease transmission. Journal of Wildlife Diseases. 2009;45(2):422–9. doi: 10.7589/0090-3558-45.2.422 19395751 10.7589/0090-3558-45.2.422

