# Correction: Quantitative approach for the risk assessment of African swine fever and Classical swine fever introduction into the United States through legal imports of pigs and swine products

**DOI:** 10.1371/journal.pone.0208065

**Published:** 2018-11-20

**Authors:** Diana María Herrera-Ibatá, Beatriz Martínez-López, Darla Quijada, Kenneth Burton, Lina Mur

In the Author Contributions section, Diana María Herrera-Ibatá (DMH-I) should be listed as one of the persons who contributed to Investigation, Resources, Software, and Writing – Review & Editing.
